# *Mycobacterium* Species Related to *M. leprae* and *M. lepromatosis* from Cows with Bovine Nodular Thelitis

**DOI:** 10.3201/eid2012.140184

**Published:** 2014-12

**Authors:** Didier Pin, Véronique Guérin-Faublée, Virginie Garreau, Franck Breysse, Oana Dumitrescu, Jean-Pierre Flandrois, Gerard Lina

**Affiliations:** VetAgro Sup Campus Vétérinaire de Lyon, Marcy l’Étoile, France (D. Pin, V. Guérin-Faublée);; Clinique Vétérinaire, Saint Bénigne, France (V. Garreau);; Centre Hospitalier Lyon Sud, Pierre Bénite, France (F. Breysse);; Université Lyon 1, Lyon, France (O. Dumitrescu, J.-P. Flandrois, G. Lina)

**Keywords:** Mycobacterium sp., Mycobacterium leprae, Mycobacterium lepromatosis, cow, dermatitis, granuloma, bovine nodular thelitis

## Abstract

Bovine nodular thelitis is a granulomatous dermatitis associated with infection with acid-fast bacteria. To identify the mycobacterium responsible for this infection, we conducted phylogenetic investigations based on partial sequencing of 6 genes. These bacteria were identified as an undescribed *Mycobacterium* species that was phylogenetically related to *M. leprae* and *M. lepromatosis*.

The genus *Mycobacterium* contains >100 species. Except for the *Mycobacterium tuberculosis* complex and *M. leprae*, which are parasitic bacteria, mycobacteria are considered saprophytic and found in soil, water, and sediments. Humans and wild and domestic animals can be infected by nontuberculous mycobacteria (NTM) from environmental sources, and several species are emerging as opportunistic pathogens in humans. NTM are often present on the skin surface after exposure to aqueous environments, and NTM skin diseases are of particular concern in humans (*1*).

Bovine nodular thelitis is a chronic and enzootic cutaneous disease that was first described in France in 1963 and then in Japan and Switzerland (*2**–­**4*). This granulomatous dermatitis is associated with acid-fast bacilli and believed to have a mycobacterial origin of infection (*2**,**4*). However, cultivation and characterization of the causal bacteria have not been successful (*3*). We used a multigene sequencing phylogenetic approach described previously (*5*) to identify the mycobacterium responsible for bovine nodular thelitis.

## The Study

**Figure 1:**
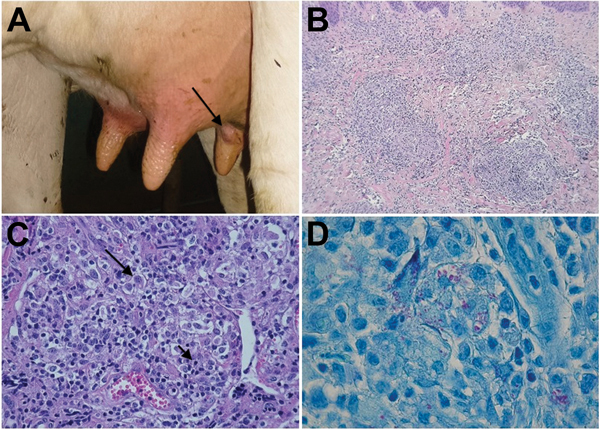
Lesions of cows with bovine nodular thelitis, Jura, France. A) Nodule on a bovine teat (arrow). B) Nodular granulomatous dermatitis, hematoxylin and eosin stained, original magnification ×100). C) Nodular granulomatous dermatitis, showing foamy macrophages (large arrow) and lymphocytes (small arrow) in an inflammatory infiltrate, original magnification ×400. D) Acid-fast bacteria. Ziehl-Neelsen stained, original magnification ×1,000.

In 2013, we visited a dairy herd in Jura, France, on 3 occasions. The herd contained ≈30 lactating cows. During physical examination of all teats, one third of the cows had lesions. In most cases, only 1 teat/cow had a lesion, which was a single, painless nodule of variable size that was localized in the dermis (Figure 1, panel A). Early-stage lesions, which had a well-demarcated indurated area, were observed only in a few cows. A few nodules evolved to ulcers, and most ulcers showed cicatrization and fibrosis.

To establish a diagnosis, we obtained 3 biopsy specimens from 3 cows (1 specimen/cow) under local anesthesia (lidocaine) from intact nodules by using a skin biopsy punch (diameter 8 mm). Each biopsy specimen was divided into 2 parts: half was placed in formalin, and half was placed in sterile 0.9% NaCl.

During histopathologic examination, superficial and deep dermis showed nodular and interstitial dermatitis (Figure 1, panel B) and an infiltrate composed predominantly of large, pale, foamy macrophages mixed with lymphocytes and few plasma cells (Figure 1, panel C). Some macrophages contained 2 or 3 nuclei and a granular cytoplasm. Multinucleated giant cells were rarely observed. There were large foci of necrosis. Ziehl-Neelsen staining indicated acid-fast bacteria deposited in clumps and resembling globi of leprosy within macrophages and necrotic foci exclusively on samples 1 and 2 (Figure 1, panel D). These observations resulted in the diagnosis of bovine nodular thelitis. Mycobacterial culture was not attempted because of its inherent difficulty (*3*). A molecular approach was used to characterize bacteria from the 3 biopsy specimens.

Biopsy specimens were washed 3 times with DNA-free sterile water, and epidermis was removed from the dermis and hypodermis to avoid contamination with cutaneous flora. The dermis and hypodermis were homogenized at 7,400 rpm for 70 s in a proteinase K solution (1 mg/mL) (Sigma, St. Louis, MO, USA) by using a MagNA Lyser (Roche Molecular Diagnostics, Mannheim, Germany), followed by incubation for 18 h at 55°C in a shaking dry bath incubator. Whole DNA was extracted from the lysed tissue by using the MagnaPure Compact Kit (Roche Molecular Diagnostics).

For the phylogenetic investigations, a multigene sequencing approach was used (*5*). Double-stranded partial sequences of the genes for 65-kDa heat shock protein (*hsp65* [*groEL2*]), the β-subunit of RNA polymerase gene (*rpoB*), superoxide dismutase (*sodA*), elongation factor Tuf (*tuf*), 16S rRNA, and transfer–messenger (tmRNA) were obtained by real-time PCR by using primers (Table) designed for this study (*rpoB*) or described previously (*5*,*6*). For each gene, chromatograms corresponding to the 2 strands of the 3 amplified products (only 2 amplified products for the 16S rRNA gene) were compared by using Staden software (http://staden.sourceforge.net/) and edited to remove ambiguous bases (*7*).

**Table:** Primers used for PCR detection of *Mycobacterium* species, Jura, France

Gene*	Sequence (5′→3′)†
16S rRNA gene (*6*)	F-TCAAAKgAATTgACgggggC
	R-ggTTACCTTgTTACgACTT
*hsp65* (*5*)	F-ACCAACgATggTgTgTCCAT
	R-CTTgTCgAACCgCATACCCT
*rpoB* (designed for this study)	2F-TCAACgggACCgAgCgTgTC
	2R-gTgTTgTCCTTCTCCAgCgT
	3F-TCAACgggACCgAgCgTgTC
	4R-gTCTCgATCgggCACATC
	9F-gTgggCACCggCATggAgTT
	9R-ATgTTCATCCgTCgCggC
	8F-ATGAAgCTgCACCACTTggT
	8R-gCCGATTCgTTgCgggACA
*sodA* (*5*)	F-AgCTTCACCACAgCAAgCACCA
	R-TCggCCAgTTCACgACgTTCCA
*tuf* (*5*)	F-CACgCCgACTACATCAAgAA
	R-gAACTgCggACggTAgTTgT
tmRNA (*5*)	F-ggggCTgAAACggTTTCgAC
	R-TggAgCTgCCgggAATCgAAC
*hsp65, heat shock protein 65; *rpoB*, β-subunit of RNA polymerase; *sodA*, superoxide dismutase A; *tuf*, elongation factor Tuf; tmRNA, transfer–messenger RNA. †F, forward; R, reverse. DNA was amplified by using a LightCycler 2.0 instrument (Roche Molecular Diagnostics, Indianapolis, IN, USA) and a ready-to-use hot start reaction mixture (TAKARA, Tokyo, Japan). For all PCRs, parameters were denaturation 95°C for 30 s; and amplification (45 cycles) at 95°C for 15 s, 65°C for 20 s, and 72°C for 40 s (slope, 2°C/s); melting curve, 0 s at 95°C, 15 s at 75°C, and 0 s at 98°C (slope, 0.05°C/s).	

For each gene, no differences were detected among 3 sequences (2 sequences for the 16S rRNA gene), and a consensus sequence was subsequently used for each gene (392 bp for *hsp65* [KJ095005], 951 bp for *rpoB* [KJ095009], 442 bp for *sodA* [KJ095006], 718 bp for *tuf* [KJ095008], 359 bp for the tmRNA gene [KJ095007], and 369 bp for the 16S rRNA gene [KJ095004]). To make phylogenetic comparisons, we selected a subset of *Mycobacterium* spp. sequences for each gene to obtain a good representation of the genomic diversity among the slow-growing mycobacteria by using data from Mignard and Flandrois (*5*). Sequences were obtained from GenBank/European Molecular Biology Laboratory/DNA Data Bank of Japan databases. The MAFFT program (http://mafft.cbrc.jp/alignment/software/) was used to align nucleotide sequences. Alignments were verified by using SeaView (http://www.molecularevolution.org/software/alignment/seaview). Divergent and ambiguously aligned blocks were removed by using Gblocks (http://molevol.cmina.csic.es/castresana/Gblocks_server.html) to ensure an accurate alignment before phylogenetic reconstructions. Phylogenies were inferred from sequences by calculating observed genetic distances by using PhyML (http://code.google.com/p/phyml/) with the general time reversible evolutionary model (*7*). *Mycobacterium setuense* was used as an outgroup because it is a species that is borderline to the slow-growing mycobacteria (*8*). Resulting tree topologies were evaluated by bootstrap analysis with 1,000 resamples.

**Figure 2:**
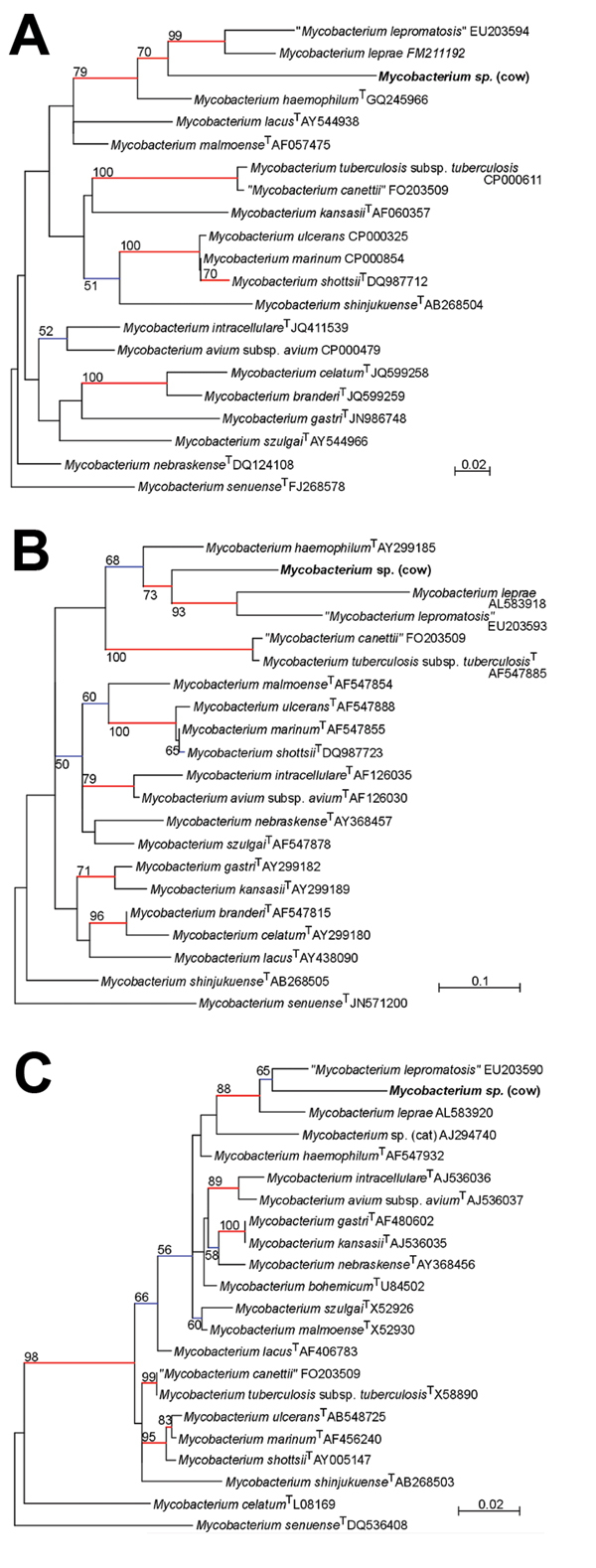
A) Phylogenetic trees based on partial A) β-subunit of RNA polymerase, B) partial heat shock protein 65 sequences, and C) partial 16S rRNA gene sequences of *Mycobacterium* spp., Jura, France. Phylogenies were inferred by using PhyML (http://code.google.com/p/phyml/) with the general time reversible evolutionary model (*7*). Trees were rooted by using *M. setuense* as an outgroup. Strains isolated in this study are indicated in bold. Values along the branches are bootstrap values (bootstrapped 1,000 times). Branches in blue indicate bootstrap values >50% and branches in red indicate bootstrap values >70%. Scale bars indicate estimated nucleotide substitutions per site.

The phylogenetic tree based on *rpoB* sequences (Figure 2, panel A) was consistent with that obtained for *hsp65* sequences (Figure 2, panel B). On the basis of *rpoB* and *hsp65* phylogenies, the unidentified organism was phylogenetically similar to *M. leprae* and *M. lepromatosis* but belonged to a clearly distinct branch. Similar to these species, it shares a common ancestor with *M. haemophilum*. The same well-sustained relationship with *M. leprae* and separation from *M. haemophilum* was inferred from *sodA*, *tuf*, and tmRNA gene phylogenies, but the lack of corresponding sequences for *M. lepromatosis* impaired this analysis.

On the basis of 16S rRNA gene phylogeny (Figure 2, panel C), the unknown organism was a member of the *M. leprae* cluster and was related to *M. lepromatosis* and an unknown feline *Mycobacterium* sp. that causes leprosy-like symptoms in cats (*9*). This organism was phylogenetically related to *M. lepromatosis*, and the 2 species constituted a separate branch from that of *M. leprae* (bootstrap value 88%). The absence of congruence between the phylogenetic trees inferred from the 16S rRNA gene and the *hsp65* and *rpoB* genes could be related to a short consensus sequence (369 bp) in a position that is not optimal for mycobacteria discrimination.

## Conclusions

Phylogenetic investigations strongly supported the conclusion that an undescribed species of the genus *Mycobacterium* that is related to *M. leprae* and *M. lepromatosis*, the causative agents of tuberculoid and lepromatous leprosy, and a diffuse form of lepromatous leprosy (*10**,**11*), respectively, was characterized in cows with bovine nodular thelitis. *M. leprae* and *M. lepromatosis* are not cultivable on artificial media. *M. haemophilum*, which is closely related to *M. leprae* and *M. lepromatosis* (*11**,**12*), is a slow-growing mycobacterium that requires hemin and a low temperature for growth (*12*). Attempts to cultivate mycobacteria from cows with bovine nodular thelitis were mostly unsuccessful and necessitated multiple samples (*3*).

*M. leprae* strains were found to be clonal (*13**,**14*). In accordance with reductive evolution of its genome (*15*), this species is an obligate parasite that infects humans and armadillos (*13*). In contrast, the environment could be a reservoir of *M. haemophilum* (*12*). The natural habitat of the causative agent of bovine nodular thelitis is unknown, and zoonotic transmission had never been observed in breeder cows.

The *M. leprae* cluster might have an animal origin. Genetic studies of multiple bovine and feline mycobacteria characterized from animals with nodular thelitis and leprosy-like syndromes, respectively, are currently in progress and should provide useful information.
